# Total Phenolic, Flavonoid, Tomatine, and Tomatidine Contents and Antioxidant and Antimicrobial Activities of Extracts of Tomato Plant

**DOI:** 10.1155/2015/284071

**Published:** 2015-11-01

**Authors:** Norma Patricia Silva-Beltrán, Saul Ruiz-Cruz, Luis Alberto Cira-Chávez, María Isabel Estrada-Alvarado, José de Jesús Ornelas-Paz, Marco Antonio López-Mata, Carmen Lizette Del-Toro-Sánchez, J. Fernando Ayala-Zavala, Enrique Márquez-Ríos

**Affiliations:** ^1^Departamento de Biotecnología y Ciencias Alimentarias, Instituto Tecnológico de Sonora, 5 de Febrero 818 Sur, 85000 Ciudad Obregon, SON, Mexico; ^2^Departamento de Ciencias de la Salud, Universidad de Sonora, Campus Cajeme, Boulevard Bordo Nuevo, Ejido Providencia, 85040 Cajeme, SON, Mexico; ^3^Centro de Investigación en Alimentación y Desarrollo A.C., Avenida Río Conchos S/N, Parque Industrial, 31570 Cuauhtémoc, CHIH, Mexico; ^4^Departamento de Investigación y Posgrado en Alimentos, Universidad de Sonora, Boulevard Luis Encinas y Rosales s/n, Colonia Centro, 83000 Hermosillo, SON, Mexico; ^5^Centro de Investigación en Alimentación y Desarrollo A.C., Carretera a la Victoria, 83000 Hermosillo, SON, Mexico

## Abstract

The purpose of this study was to evaluate the antioxidant and antimicrobial properties of extracts of different fractions of two tomato plant cultivars. The stems, roots, leaves, and whole-plant fractions were evaluated. Tomatine and tomatidine were identified by HPLC-DAD. The leaf extracts from the two varieties showed the highest flavonoids, chlorophyll, carotenoids, and total phenolics contents and the highest antioxidant activity determined by DPPH, ABTS, and ORAC. A positive correlation was observed between the antioxidant capacities of the extracts and the total phenolic, flavonoid, and chlorophyll contents. The Pitenza variety extracts inhibited the growth of pathogens such as *E. coli* O157:H7, *Salmonella *Typhimurium*, Staphylococcus aureus*, and *Listeria ivanovii*, yielding inhibition halos of 8.0 to 12.9 mm in diameter and MIC values of 12.5 to 3.125 mg/mL. These results suggest that tomato plant shows well potential as sources of various bioactive compounds, antioxidants, and antimicrobials.

## 1. Introduction

Tomato plants belong to the Solanaceae family and are known as a good source of phenolic compounds, pigments, antioxidants, and other nutrients in the human diet [1]. Substantial quantities of tomatoes are processed into juices and purees, which generate wastes. In the agribusiness sector, tomato plants are discarded after harvesting because they are considered worthless for industrial processes. The remaining plant materials are typically used as an unbalanced food for feeding livestock (mainly cattle), without any apparent economic benefit for the tomato producers.

Some reports have shown that tomato fruit extracts exhibit antimicrobial and anticancer properties [2, 3]. The phenolic contents of tomato fruits have been correlated with their antioxidant capacity. These compounds also prevent oxidative changes in cells by reducing the levels of free radicals [4, 5], and epidemiological reports suggest a direct correlation between the antioxidant capacity of tomatoes and a decreased risk of developing cardiovascular disease and cancer [6]. In addition to these properties, tomato byproducts, such as the seeds, represent an attractive source of fiber [7] that also shows antimicrobial activities [8]. Tomato plants also possess bioactive components with pharmacological and nutritional properties [9].

Plant tissues are a focus of study because of the biologically active compounds that have been isolated from them. Different parts of plants are rich in various types of bioactive compounds, many of which are discarded as byproducts by the food industry, which utilizes only certain parts of the fruit. The cuticles, peels, pulp, seeds, stems, and leaves of plants are potential sources of antimicrobial, antiviral, and antioxidant compounds, such as phenols, flavonoids, and vitamins, among others. In the last decade, the available literature addressing the antioxidant and antimicrobial activities of plant byproduct has increased considerably, particularly concerning grape seed extracts and olive wastes [10].

Currently, the trend in the agribusiness sector is to recover, evaluate, and find better uses for all their byproducts such as, peels, seeds, stems, and leaves. Tomato crop byproducts contain bioactive substances that could be potential sources of antimicrobial, antiviral, and antioxidant compounds, giving them economic value in the food industry. In this context, the aim of this study was to investigate the composition (steroidic alkaloids, total flavonoids, phenols, carotenoids, and chlorophyll) and the antioxidant and antimicrobial capacities of extracts from two tomato cultivars (Pitenza and Floradade), which could be useful in the selection of materials for the production of bioactive compounds and nutraceuticals.

## 2. Material and Methods

### 2.1. Chemicals

Potassium persulfate, 2,2-azino-bis(3-ethylbenzothiazoline-6-sulfonic acid) diammonium salt (ABTS), 2,2-diphenyl-1-picrylhydrazyl (DPPH), Folin-Ciocalteu reagent, 6-hydroxy-2, 5, 7, 8-tetramethylchroman-2-carboxylic acid (Trolox), sodium carbonate, gallic acid, quercetin, amoxicillin, tomatine, tomatidine, and acetone were purchased from the Sigma Chemical Co. (St. Louis, MO, USA). Mueller-Hinton agar, DMSO (dimethyl sulfoxide), and all other nonspecified reagents and solvents were purchased from J.T. Baker (Baker-Mallinckrodt, México).

### 2.2. Plant Material

In this study, we used residues of tomato plants (*Lycopersicon esculentum*) of the Pitenza and Floradade varieties, which were obtained from greenhouses in the Yaqui Valley in Sonora, México. Twenty sample fresh plants were collected for each plant variety and were washed with distilled water. The parts of the plants that were used to obtain extracts were the roots, stems, leaves, and whole plants.

### 2.3. Bacterial Strains and Growth Conditions

*Escherichia coli *O157:H7 (ATCC 43890),* Staphylococcus aureus *(ATCC 65384),* Salmonella *Typhimurium (ATCC 14028), and* Listeria ivanovii* (ATCC 19119) were employed in the experiments. These strains were maintained in tryptone soy broth (TSB) containing glycerol (20%) at −40°C until use. A loopful of bacteria was transferred to 10 mL of TSB and incubated at 37°C overnight. A loopful of that culture was then transferred to TSB, and the culture was grown at 37°C until reaching the desired number of colony-forming units per mL (cfu/mL) for use in inhibitory assays.

### 2.4. Preparation of Extracts

Stem, leaf, root, and whole-plant fractions were subjected to drying in a vacuum oven for 24 h, which was conducted at 45°C except in the case of the roots and stems, which were dried at 60°C. The dried materials were pulverized and passed through a Number 20 sieve (WS Tyler). A 35 g aliquot of the sieved sample was then mixed with a solution of ethanol and 5% acetic acid (95 : 5 ratio), and maceration was carried out via constant stirring for 72 h on a stir plate in complete darkness at room temperature. The sample was subsequently vacuum filtered through Whatman Number 1 paper, and the residue was extracted to exhaustion with the acid-ethanol mixture via sonication for 20 min (Branson 3510). This sample was filtered, and the two solvents were mixed and evaporated using a rotatory evaporator (Buchi Heating Bath B-490, Buchi Rotvapor R-200). Finally, the extract was lyophilized for 48 h (Freeze zone 4.5, Labconco), and the dried extracts were maintained at −20°C for subsequent analysis. All the extractions were performed in triplicate and the glycoalkaloids, total phenolic, flavonoids, chlorophyll, antioxidant, and antibacterial activity were measured.

### 2.5. Measurement of Glycoalkaloids Compounds by HPLC

The presence of tomatine and tomatidine was measured using adaptations from the methods proposed by [11, 12], where the extract was dissolved in 0.2 N HCl and the glycoalkaloids precipitated with 5% NH_4_OH until a pH = 10. This solution was centrifuged at 10,000 g for 10 min at 4°C. The washing was repeated. The supernatant was evaporated at 45°C and the pellet was dissolved in MeOH and filtered through a 0.45 *μ*m membrane and volume of 10 *μ*L was injected into the high-performance liquid chromatography (HPLC) water 2690 diode array detector equipped with degassing system. The specific settings of the HPLC were as follows: (a) column, Zorbax SB-C18 (4.6 mm ID × 250 mm Zorbax column with a 5 *μ*m particle size); (b) uv-visible detector (waters 717 plus); (c) the absorbance being recorded at 200 nm; and (d) a mobile phase, MeOH, which was utilized. A minimum of five min was kept between runs to allow for equilibrium. The glycoalkaloid content was calculated using standard curves and was expressed as mg/ge. Each extract was analyzed three times.

### 2.6. Total Phenolics

The concentration of total phenolic content was determined using the Folin-Ciocalteu method described by Magalhães et al. [13], with some modifications. The extracts were dissolved in absolute methanol and later 15 *μ*L of the extract were mixed with 750 *μ*L of 1 N Folin-Ciocalteu reagent (1 : 10). After 5 min at room temperature, 60 *μ*L of Na_2_CO_3_ (7.5%) was added to the extracts. Following an incubation for 30 min at room temperature, the absorbance was read at 765 nm using a multimode microplate reader Fluostar Omega (BMG Labtech, Chicago, IL, USA). The concentration of total phenolic compounds was calculated using a standard curve of gallic acid equivalents (GAE) and expressed as milligrams per gram of extract (mg GAE/ge). Analyses were performed in triplicate per each extract.

### 2.7. Total Flavonoids

The flavonoid content was determined based on the method described by Chen at al. [14], with slight modifications. The extracts were dissolved in absolute methanol. In a 2 mL Eppendorf tube, 100 *μ*L of a sample was mixed with 430 *μ*L of 5% NaNO_2_, followed by incubation for 5 min. After incubation, 30 *μ*L of AlCl_3_ (10%) and 440 *μ*L of NaOH (1 mol/L) were added to the reaction mixture, and the absorbance was read at 496 nm with a multimode microplate reader Fluostar Omega spectrophotometer (BMG Labtech, Chicago, IL, USA), using quercetin as the standard. The results were expressed as mg of quercetin equivalents (QE) per g of extract (mg QE/ge).

### 2.8. Total Chlorophyll

A 0.2 g sample was extracted 3 times with 80% acetone until the green pigments disappeared. The final mixture was filtered, and the supernatants were combined, and the volume was then brought to 25 mL. The absorbance was measured at 663, 652, 645, and 470 nm using a UV-VIS spectrophotometer (Genesis 20). The chlorophyll contents were expressed as mg/g of extract (ge) according to the following equations [15]:

(1) Chlorophyll  a=12.7×A663−2.7×A645,Chlorophyll  b=22.9×A645−2.7×⁡A663,Total  chlorophyll=27.8×A652,Total  carotenoids=1,000×A470−1.63 Chlorophyll  a×104.96 Chlorophyll  b221.

### 2.9. Antioxidant Activity

#### 2.9.1. ABTS Assay

This assay was conducted according to Toor et al. [16], with some modifications. ABTS radical cations were generated in a mixture of 5 mL of a 7 mmol ABTS solution and 88 *μ*L of a 0.139 mmol K_2_S_2_O_8_ solution. The extracts were diluted with absolute ethanol. The reaction mixture was placed in a microplate with 5 *μ*L of extract in the presence of 245 *μ*L of ABTS solution. The absorbance was read at 734 nm after the initial mixing (Absi) and every minute thereafter for 7 min (Absf) using a microplate reader Fluostar Omega spectrophotometer (BMG Labtech, Chicago, IL, USA). All determinations were carried out in triplicate. The TEAC value was expressed as Trolox equivalents (mmol TE) per g of extract (ge).

#### 2.9.2. DPPH Assay

This assay was conducted according to Chandra and Ramalingam [5], with some modifications. An aliquot of 280 *μ*L of a solution of DPPH radicals (0.025 mg/mL in methanol) was mixed with 20 *μ*L/mL of each extract diluted in ethanol and shaken. The reaction was allowed to proceed at room temperature for 30 min, and the absorbance was measured at 515 nm using a Fluostar Omega spectrophotometer (BMG Labtech, Chicago, IL, USA) microplate reader device. A control reaction mixture was prepared without any extract. The antioxidant activity was calculated using a Trolox calibration curve and expressed as Trolox equivalents per g of extract (mmol TE/ge).

#### 2.9.3. ORAC Assay

The ORAC was determined according to López-Cobo et al. [17]. The AAPH reagent was used as the peroxyl radical generator, fluorescein (FL) as the fluorescent indicator, and Trolox as the standard. The reaction mixture contained 100 *μ*L of ethanol solution of extract, 1.65 *μ*L of phosphate buffer (10 nM, pH 7.4), 150 *μ*L of AAPH (0.8 M), and 100 *μ*L of FL (0.106 *μ*M). Phosphate buffer was used as the blank. The samples were preincubated at 37°C for 15 min, and AAPH was then added. Fluorescence was monitored at 484–515 nm using a microplate reader Fluostar Omega spectrophotometer (BMG Labtech, Chicago, IL, USA). The final ORAC values were expressed as Trolox equivalents per g of extract (mmol TE/ge).

### 2.10. Antibacterial Activity

Antimicrobial activity was evaluated by measuring bacterial growth inhibition zones, as described by Jordán et al. [18], with some modifications. Mueller-Hinton agar plates were inoculated with 100 *μ*L of a freshly prepared bacterial suspension (10^8^ CFU/mL). Each extract was diluted with 10% dimethyl sulfoxide (DMSO) and sterilized via filtration through a 0.45 *μ*m membrane filter. A 40 *μ*L sample of each extract was loaded onto sterile filter paper discs (6 mm in diameter, Whatman Number 1), which were subsequently placed on top of the agar plates. The plates were left for 30 min at room temperature and then incubated at 37°C for 24 h, and the growth inhibition halos were measured. These assays were conducted in triplicate, and the mean values were calculated. Negative control treatments consisted of discs impregnated with DMSO (10%). Amoxicillin (50 *μ*g/mL) was used as the positive control. The minimum inhibitory concentration (MIC) was determined via microdilution according Taveira et al. [8], with some modifications. The tested bacteria were inoculated into solutions of the tomato plant extracts in 96-well microtitration plates at a concentration of 10^7^ CFU/mL. After incubation at 37°C for 24 h, the MIC was determined as the lowest concentration of extract at which no metabolic activity was observed in the tested bacteria. Turbidity indicated growth of the microorganisms. Amoxicillin served as the positive control.

### 2.11. Statistical Analysis

The results are expressed as mean values ± SD, and statistical significance was set at the 5% level (*P* < 0.05). The Tukey-Kramer multiple comparison test was applied. Correlation analysis was performed to determine the relationships between the antioxidant properties of the extracts and the phytochemical compounds they contained based on the Pearson correlation coefficient and significance level (*P*). Statgraphics Plus v. 5.0 software was used.

## 3. Results and Discussion

### 3.1. Alkaloids Composition

The glycoalkaloids are metabolites that protect of plants against including insects, fungi, bacteria, and viruses. In addition, they present a variety of pharmacological and nutritional properties in animals and humans [1, 11]. The leafs of the two varieties of tomato plant evaluated in this study showed the highest contents of tomatine and tomatidine with values of 4.940 and 0.820 and 2.430 and 0.225 mg/ge by Pitenza and Floradade, respectively (Table 1). However these values are lower to those reported by Taveira et al. [1] in leaves extracts. Tomatine and tomatidine were not detected in root extracts in neither of the cultivars. This finding coincides with the results reported by Kozukue et al. [12] in the roots of tomato plant. The reason for this discrepancy between leaves and roots could be that the glycoalkaloids are concurrently synthesized and then degraded during plant maturation [12].

### 3.2. Phenolic, Flavonoid, Chlorophyll, and Carotenoid Composition

**Figure 1:**
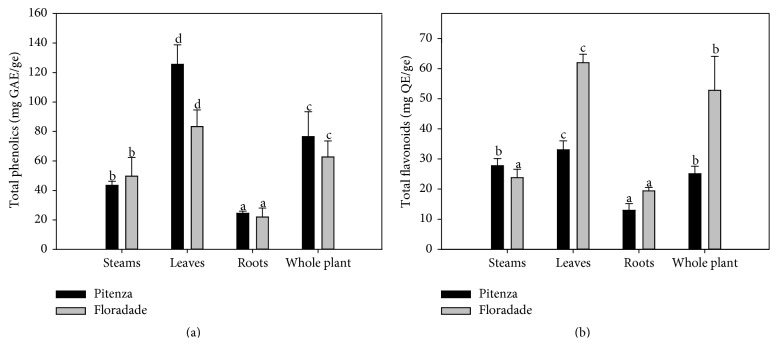
Total phenolic acids (a) and total flavonoids (b) in extracts of tomato plants. The data are mean values ± SD from at least three determinations. The mean values represented by the bars for each type of extract that are indicated with a different letter are significantly different (*P* ≤ 0.05).

Plants contain a large variety of phenolic derivatives. These compounds are essential for plant growth and reproduction. In addition, phenolic compounds are natural antioxidants that may occur in all parts of the plant and function as antibiotics and natural pesticides [19].  Figure 1(a) shows the total phenol contents of the examined sections from the Pitenza and Floradade plant tomato varieties. The leaf extracts of the two varieties exhibited the highest phenolic contents with values of 125.5 and 83.35 mg GAE/ge by Pitenza and Floradade, respectively. All of the Pitenza plant sections displayed significantly (*P* < 0.05) higher phenolic contents compared to the Floradade sections. In contrast, the root extracts displayed the lowest phenolic contents among both the Pitenza and Floradade plant sections (24.5 and 21.98 mg GAE/ge, resp.), showing that roots are not an important reservoir of this type of compound.

Studies in tomato fruit byproducts reported phenolic levels of 44.18 and 20.94 mg GAE/100 g in tomato skin and seeds, respectively. Peschel et al. [20] observed a content of 61 mg GAE/g dry extract in tomato peels. Rivero et al. [21] studied the levels of phenols in tomatoes subjected to stress and reported total phenol values of 2.66 to 3.55 mg caffeic acid equivalents per g. All of the previously reported values for tomatoes were lower than those obtained in this study. Therefore, our results suggest that tomato plant extracts contain higher levels of phenols than those found in the fruit. This phenomenon most likely occurs because the stress caused by UV radiation and other factors results in the accumulation of phenolic compounds in the vacuoles of plant dermal tissues, as such compounds are used as a defense against these factors [5]. Another reason for this discrepancy could be that the extracts evaluated in this study were obtained from plants that were in a mature physiological state. Toor et al. [16] reported that high temperatures and light exposure stimulate the production of phenolic acids and other flavonoids and that heat stress in tomato plants increases the activity of phenylalanine ammonia-lyase (PAL) as well as the total phenol and* o*-diphenol contents and induces the accumulation of phenolics by activating their biosynthesis as well as inhibiting their oxidation. In this study, the leaf extracts from the Pitenza and Floradade varieties displayed contents of 125.501 and 83.356 mg GAE/ge, respectively. Rivero et al. [21] reported a similar behavior in the leaves of the tomato plant, possibly because these parts of the plant are exposed to more direct light and UV radiation than other plant sections and therefore exhibit greater amounts of phenolic compounds. Cervilla et al. [22] demonstrated that increases in polyphenol levels in tomato leaves and the accumulation of soluble phenols in tomato plants are mechanisms induced in response to stress stimulated by boron. The analysis of total phenols in the extracts from different plant parts (Figure 1(a)) revealed significant differences (*P* < 0.05) among the extracts. Many of the phytochemicals in plants can be detected at different concentrations because the amount and composition of secondary metabolites are not constant, and their concentration depends on the tissue type and the age of the plant [23]. Recently we reported the characterization of the phenolics compounds from the tomato plant and identified 6 compounds, of which gallic acid, chlorogenic acid, and rutin were the predominant components and the leaves of the plant showed high accumulation of this compound [24].

In relation to flavonoid compounds, Toor et al. [16] reported that the phenolic contents of tomato plants consist of hydroxycinnamic acid and flavonoids, located mainly in the leaves. Here, we found that the leaf extracts exhibited the highest values among the examined plant sections, with values of 33.028 and 61.96 mg QE/ge being obtained for the Pitenza and Floradade varieties, respectively (Figure 1(b)). We also found that the concentration of flavonoids was significantly affected by the variety of the tomato plants. This finding is similar to those reported by Martínez-Valverde et al. [25]. These authors observed that the flavonoid concentration was affected by the tomato fruit variety and that this variation could be due to genetic differences as well as different environmental stress conditions and agricultural practices that affect the chemical composition of plants. Slimestad and Verheul [26] detected quercetin and rutin in the leaves of two tomato cultivars. Our results showed that the leaf and stem extracts had high contents of flavonoids expressed as quercetin. Future studies should focus on phytochemicals such as rutin and quercetin in tomato plant residues, as these compounds represent the main flavonoids present in tomato plants and act as potent antioxidants with beneficial health effects [27].

Tomato plants synthesize metabolites and pigments, such as chlorophyll and carotenoids, that benefit human nutrition and health, and some research has indicated that these phytochemicals are strongly affected by the maturity of the plant and that they prevent photooxidation [28].  Table 2 provides the contents of chlorophyll a, chlorophyll b, and total chlorophyll in the examined extracts, which varied significantly (*P* < 0.05). The root extracts presented the lowest contents in both varieties. In contrast, the Pitenza and Floradade leaf extracts exhibited the highest contents, followed by the whole-plant and stem extracts. These aerial parts of the plants are the anatomical parts that are most exposed to sunlight. Lumpkin [29] reported that the contents of chlorophyll and carotenoids in tomato plants are strongly affected by the incidence of light and that the concentrations of these metabolites are increased by exposure to light. The content of chlorophyll a was higher in the leaf extract from the Floradade variety. These results are similar to those reported by Ferruzzi et al. [30], who found that plant chlorophyll a is less affected by and exhibits a greater ability to inhibit DPPH and ABTS radicals compared to chlorophyll b.

The total carotenoid concentrations in the different parts of the plants are reported in Table 2. The leaf extracts from the two varieties of tomato showed the highest concentrations. However, the concentration in the Floradade leaf extract was significantly (*P* < 0.05) higher (5.354 mg/ge) compared to that of Pitenza (1.221 mg/ge). Nevertheless, Pitenza extract showed the highest level of total phenols. In agreement with previous studies on phytochemicals, the roots presented the lowest carotenoid content. The carotenoid content in all parts of the plant was significantly higher for the Floradade compared to the Pitenza variety (*P* < 0.05). This result suggests that the carotenoid composition can vary depending based on the material (raw or not) and the maturity of plant, both of which result in variation of the degradation rate of carotenoids [31].

### 3.3. Antioxidant Activity

**Figure 2:**
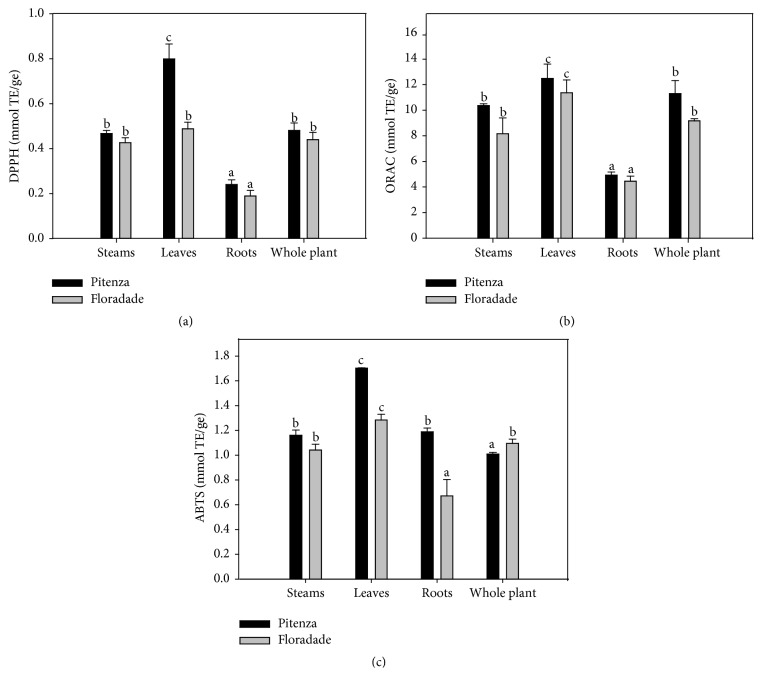
Antioxidant capacity measured by DPPH (a), ORAC (b), and ABTS (c) assay. Data are means of at least three determinations. Mean values in each bar followed by different letter each extracts of tomato plant.

An antioxidant is any substance that, when present at low concentrations, significantly delays or prevents the oxidation of cellular components, such as proteins, lipids, carbohydrates, and DNA [19]. We evaluated the antioxidant activity of the extracts as DPPH, ABTS, and peroxyl radicals in ORAC assays and the results are reported as mmol TE/ge (Figure 2). The antioxidant activities of the extracts from all parts of the Pitenza variety plants determined using these three methods were higher compared to those of the Floradade variety. The extracts from leaves of the two varieties of tomato presented the highest TE values, followed by the stem and whole-plant extracts, which showed no significant differences. The antioxidant activity of the root extracts was the lowest among all of the samples included in this study. This low antioxidant capacity could be related to the low contents of phytochemicals such as phenolics and flavonoids in these extracts (Figures 1(a) and 1(b)). Another explanation could be that the roots of some varieties of tomato plant are not subject to the same levels of stress as other parts of the plant, showing a decreased response to antioxidant enzymes, such as catalase (CAT), superoxide dismutase (SOD), and ascorbate peroxidase (APX) [32, 33].

The overall results of the present work revealed that the extracts from the leaves of the two varieties of tomato exerted high antioxidant activity. The leaf extract from the Pitenza variety showed higher activity, displaying DPPH, ABTS, and ORAC values of 0.798, 1.702, and 13.489 mmol TE/ge, respectively (Figures 2(a), 2(b), and 2(c)). The higher activities observed in the leaves could represent a defense mechanism in the plant resulting from being exposed to the high temperatures that occur in the geographical area from which the samples were obtained. Previous studies have shown that when* Festuca arundinacea* and tomato leaves are heat treated, their antioxidant activities are increased up to 10-fold, which supports the results of the present study [21, 34].

### 3.4. Correlation between Phytochemical Contents and Antioxidant Capacity

It has been reported that the antioxidant capacity of a plant extract is correlated with its phenolic content [35]. To determine the correlation between the phytochemical contents of the extracts and their antioxidant capacity, a Pearson correlation analysis was performed. It was clear that the phenolic content of the extracts showed a satisfactory and significant correlation with the DPPH, ABTS, and ORAC values (*P* < 0.05), with *r* values of 0.944, 0.787, and 0.870 being obtained, respectively (Table 3). These results indicated that the phenolic compounds in the tomato byproducts contributed positively to their antioxidant capacity by reducing the levels of free radicals. Similar results were reported by Spencer et al. [35], who found a correlation of *r* = 0.732 for the extracts of 37 different varieties of tomato. Toor et al. [16] reported significant correlations between the total phenolic contents and antioxidant capacities of hydrophilic and lipophilic extracts of tomatoes, with a value of *r* = 0.84 being observed. In contrast, as shown in Table 3, there was no positive correlation between the carotenoid contents and antioxidant capacities of these extracts. However, the correlations between the total flavonoid contents and DPPH, TEAC, and ORAC values were moderately strong, presenting *r* values of 0.824, 0.584, and 0.895, respectively. Tomato plant is rich in polyphenols compounds (flavonoids and hydroxycinnamic acids), such as chlorogenic, caffeic, and ferulic acid, and rutin flavonoid [16, 24], and these compounds have phenolics rings and hydroxyl groups that act trapping the free radical and inhibiting the generation of reactive oxygen species. Several flavonoids have been identified from tomato plant and the most predominant compound is quercetin-3-rutinoside (rutin). Some authors have described that rutin can neutralize free radicals by transferring and electron donation or inhibiting the activity of enzymes or lipids involved in the production of free radicals [36].

On the other hand, the correlation coefficients between the total chlorophyll contents and the assayed antioxidant capacities were higher than for the total phenolic compounds. These results suggest that, in addition to the total content of phenolic compound metabolites, chlorophyll a and chlorophyll b and total chlorophyll exhibit high free radical-scavenging activity in the extracts from the tomato plants. This behavior was similar to that reported by Ferruzi et al. [30], who found that chlorophyll has the ability to inhibit DPPH and ABTS free radicals. It has also been reported that chlorophyll shows antioxidant activity, and it has been suggested that chlorophyll reduces free radicals by acting as a H^+^ donor to break the chain reaction that causes cellular oxidation [37].

### 3.5. Antibacterial Activity

The antibacterial activities of the tomato plant extracts are shown in Table 4. The Pitenza variety extracts displayed antibacterial activity against* E. coli* O157:H7,* Salmonella* Typhimurium,* Staphylococcus aureus*, and* Listeria ivanovii*. The Pitenza extracts exhibited higher antibacterial activity compared to the Floradade extracts. The Floradade root extract showed no activity. In both varieties, the leaf extracts showed the highest antimicrobial activity, followed by the stem and whole-plant extracts. The trend of these results resembles that shown in Figure 1(a) for the total phenolic contents. Thus, the increased concentration of phenolic compounds implies an increased antimicrobial activity. Our results are similar to those reported by Smirnova et al. [38], who observed that high levels of polyphenols were positively correlated with antimicrobial activity against* E. coli*. Bashan et al. [39] evaluated the effects of* Pseudomonas syringae* on the phenolic extracts of tomato leaves and observed that the increase in the phenolic compound level is affected by the degree of microbiological contamination and serves as a response mechanism when the leaves are attacked by microorganisms. The latter finding supports the results of the present work, which showed that polyphenols will be responsible for the antimicrobial activity observed in the screened extracts of tomato plants. Some authors have described that hydroxyl groups of polyphenols cause inhibitory action in microorganisms, and these groups can interact with the cell membrane of bacteria to destroy the membrane composition and cause the loss of cellular components. Additionally, it has been reported that these OH groups can act in the active site of enzymes and damage the metabolic processes of microorganisms. Moreover, some studies have been found that the position of OH group in the aromatic ring of polyphenols as well as the length of the saturated side chain can also increase in the antimicrobial activity [40, 41]. In addition, the aldehyde structure of some phenolics compounds is associated with the double bond of carbon-carbon, which has high electronegativity, and can probably interfere with electron transfer of proteins and nucleic acids and increase its antimicrobial activity [42].

On the other hand, we evaluated the presence of glycoalkaloids in the extracts (Table 1), because they also could be an indicator of antimicrobial activity. Ours results showed that tomatine and tomatidine in leaves extracts showed the highest levels 5.4 and 2.5 *μ*M/ge for Pitenza and Floradade, respectively (data not shown). Consequently, the presence of these glycoalkaloids could increase the susceptibility of strains evaluated. Previous reports showed that tomatine has an inhibitory activity and tomatidine presents bacteriostatic activity against* S. aureus *SCV_s_ [43]. Although the steroidal alkaloids represent only 0.5% in leaves extracts and total phenols represent 14%, these findings are not discarding a synergistic effect with phenolic compounds. Some authors have reported that tomatidine isolated from tomato plant has the ability to act in synergic form and potentiate the effect of aminoglycoside antibiotics, and it plays an important role against pathogens such as* S. aureus* [44]. Milner et al. [45] reported that the antimicrobial activity of tomatidine can be correlated with the composition of the carbohydrate side chain and the nature of the aglycone moiety of glycoalkaloid. Furthermore, it has been reported that the fraction spiroaminoketal of tomatidine is important for antimicrobial activity [46].

The results of MICs showed that the tomato plant extracts exerted different degrees of growth inhibition against the food-borne bacterial strains (Table 4). In general, it was observed that the leaf extracts from the Pitenza cultivar exhibited the highest antibacterial activity and MIC value (3.125 mg/mL). The extracts caused growth inhibition in Gram (+) strains associated with MIC values of 12.5 to 3.125 mg/mL, whereas* S*. Typhimurium and* E. coli* demonstrated greater resistance to the extracts, resulting in MIC values of 25 to 12.5 mg/mL. In contrast, the activity of amoxicillin was not comparable with that of any of the extract studied. Hence, the extracts derived from the leaves of the two cultivars (Pitenza and Floradade) represent potential antimicrobial preservatives for use in foods or in pharmaceutical industries. It is also important to note that most of the available scientific reports are focused on the antimicrobial activity of tomato fruit extracts [8].

## 4. Conclusions

A wide range of antioxidant activities and antimicrobial properties were confirmed among the examined extracts of tomato plants. The extracts from the Pitenza variety exhibited the highest antioxidant and antimicrobial activities. Furthermore, the chlorophyll and flavonoids in the leaf extracts were found to play an important role in their antioxidant activity. These results suggest that extracts of tomato plant could be used as natural sources of antioxidant and antimicrobial compounds.

## Figures and Tables

**Table 1:** Glycoalkaloids composition of tomato plant extracts (mg/ge).

Plant fractions	Tomatine^a^	Tomatidine^b^
Pitenza		
Steam	0.243 ± 0.07^a^	n.d
Leaves	4.940 ± 0.31^c^	0.820 ± 0.07^b^
Root	n.d	n.d
Whole plant	1.460 ± 0.5^b^	0.240 ± 0.084^a^
Floradade		
Steam	0.238 ± 0.01^a^	n.d
Leaves	2.430 ± 0.467^c^	0.225 ± 0.07^b^
Root	n.d	n.d
Whole plant	0.660 ± 0.007^b^	0.170 ± 0.002^a^

All values are mean ± standard deviation (*n* = 3). Means with different letters within a column are significantly different (*P* < 0.05). n.d: not detected.

**Table 2:** Total chlorophyll, total carotenoids, and chlorophyll a and chlorophyll b contents in tomato plant extracts.

Sample	Chlorophyll a	Chlorophyll b	Total Chlorophyll	Total Carotenoids
	mg/ge			
Pitenza				
Steam	5.542 ± 0.005^d^	4.113 ± 0.082^d^	11.028 ± 0.038^d^	0.409 ± 0.130^c^
Leaves	4.469 ± 0.002^c^	1.512 ± 0.004^b^	5.361 ± 0.010^c^	1.221 ± 0.025^d^
Root	1.367 ± 0.003^a^	0.024 ± 0.001^a^	1.679 ± 0.008^a^	0.360 ± 0.007^a^
Whole plant	2.896 ± 0.002^b^	1.745 ± 0.002^c^	4.626 ± 0.009^b^	0.211 ± .002^b^
Floradade				
Steam	3.496 ± 0.036^c^	1.526 ± 0.031^b^	5.125 ± 0.044^b^	1.772 ± 0.030^b^
Leaves	5.362 ± 0.017^d^	3.846 ± 0.002^d^	8.348 ± 0.101^c^	5.354 ± 0.025^d^
Root	0.120 ± 0.004^a^	0.381 ± 0.011^a^	0.460 ± 0.003^a^	0.493 ± 0.001^a^
Whole plant	2.243 ± 0.006^b^	2.495 ± 0.507^c^	4.491 ± 0.902^b^	2.845 ± 0.219^c^

All values are mean ± standard deviation (*n* = 3). Means with different letters within a column are significantly different (*P* < 0.05).

**Table 3:** Pearson correlation coefficients (*r*) and level (*P*) of linear regression among different antioxidants and metabolites.

Evaluation	Total phenols		Total flavonoids		Total carotenoids		Total chlorophyll	
	*r* ^*a*^	*P*	*r* ^*a*^	*P*	*r* ^*a*^	*P*	*r* ^*a*^	*P*
DPPH	0.944	0.0004	0.842	0.0087	0.273	0.5060	0.940	0.0005
ABTS	0.783	0.0203	0.586	0.0264	0.291	0.4837	0.873	0.0045
ORAC	0.870	0.0049	0.815	0.0026	0.354	0.3883	0.901	0.0030

*r* ^*a*^ between 0.450 and 0.900 indicates a moderately strong correlation, and *r* ^*a*^ between 0.900 and 1.000 indicates a strong correlation between variables.

**Table 4:** Antibacterial activity of tomato plant extracts.

Sample	Bacterial species							
	*S*. Typhimurium		*E. coli* O157:H7		*S. aureus*		*L. ivanovii*	
	Zone of inhibition^a^	MIC^b^	Zone of inhibition^a^	MIC^b^	Zone of inhibition^a^	MIC^b^	Zone of inhibition^a^	MIC^b^
Pitenza								
Steam	10.0 ± 0.8	>25	8.6 ± 0.5	12.5	10.3 ± 1.0	12.50	11.1 ± 0.1	6.25
Leaves	10.8 ± 0.6	12.5	9.4 ± 0.9	12.5	11.3 ± 0.4	3.125	12.9 ± 0.2	3.125
Root	8.7 ± 0.5	>25	8.3 ± 1.0	>25	9.0 ± 0.8	>25	8.0 ± 0.0	>25
Whole plant	8.0 ± 0.5	>25	8.2 ± 0.4	>25	9.9 ± 0.4	>25	8.9 ± 0.5	>25
Floradade								
Steam	n.a	n.d	n.a	n.d	10.3 ± 1.0	12.0	n.a	n.d
Leaves	10.0 ± 0.2	>25	9.4 ± 0.9	>25	9.3 ± 1.2	12.5	9.0 ± 0.0	12.5
Root	n.a	n.d	n.a	n.d	n.a	n.d	n.a	n.d
Whole plant	n.a	n.d	7.7 ± 0.3	>25	8.2 ± 0.9	>25	n.a	n.d
Amoxycillin^c^	18.1 ± 0.1	0.07	21.9 ± 0.5	0.05	22.4 ± 0.1	0.05	24.7 ± 0.5	0.03
DMSO 10%^d^	n.a	n.d	n.a	n.d	n.a	n.d	n.a	n.d

All values are means of three replications; n.a: no activity; n.d: no determined; ^a^Diameter of inhibition expressed (mm); ^b^Minimal Inhibitory concentration (mg of extract/mL); ^c^Positive Control; ^d^Negative control.
